# Participant characteristics associated with withdrawal from a large randomized trial of spermicide effectiveness

**DOI:** 10.1186/1471-2288-4-23

**Published:** 2004-10-01

**Authors:** Elizabeth G Raymond, Pai Lien Chen, Bosny Pierre-Louis, Joanne Luoto, Kurt T Barnhart, Lynn Bradley, Mitchell D Creinin, Alfred Poindexter, Livia Wan, Mark Martens, Robert Schenken, Cate F Nicholas, Richard Blackwell

**Affiliations:** 1 Family Health International, Research Triangle Park, NC, USA; 2 National Institute of Child Health and Human Development, Rockville, MD, USA; 3 Department of Obstetrics and Gynecology and Center for Clinical Epidemiology and Biostatistics, University of Pennsylvania Medical Center, Philadelphia, PA, USA; 4 Department of Reproductive Medicine, Johns Hopkins Community Physicians, Baltimore, MD, USA; 5 University of Pittsburgh and the Magee-Womens Research Institute, Pittsburgh, PA, USA; 6 Department of, Baylor College of Medicine, Houston, TX, USA; 7 Department of Obstetrics and Gynecology, New York University School of Medicine, New York, NY, USA; 8 Minneapolis Medical Research Foundation, Hennepin County Medical Center, Minneapolis, MN, USA (Current affiliation: Department of Obstetrics and Gynecology, University of Oklahoma College of Medicine, Tulsa, OK, USA); 9 Department of Obstetrics and Gynecology, The University of Texas Health Science Center at San Antonio, San Antonio, TX, USA; 10 Vermont Women's Choice Program of Planned Parenthood, Burlington, VT, USA; 11 Current affiliation: University of Vermont College of Medicine, Burlington, VT, USA; 12 Department of Obstetrics and Gynecology, University of Alabama at Birmingham, Birmingham, AL, USA

## Abstract

**Background:**

In most recent large efficacy trials of barrier contraceptive methods, a high proportion of participants withdrew before the intended end of follow-up. The objective of this analysis was to explore characteristics of participants who failed to complete seven months of planned participation in a trial of spermicide efficacy.

**Methods:**

Trial participants were expected to use the assigned spermicide for contraception for 7 months or until pregnancy occurred. In bivariable and multivariable analyses, we assessed the associations between failure to complete the trial and 17 pre-specified baseline characteristics. In addition, among women who participated for at least 6 weeks, we evaluated the relationships between failure to complete, various features of their first 6 weeks of experience with the spermicide, and characteristics of the study centers and population.

**Results:**

Of the 1514 participants in this analysis, 635 (42%) failed to complete the study for reasons other than pregnancy. Women were significantly less likely to complete if they were younger or unmarried, had intercourse at least 8 times per month, or were enrolled at a university center or at a center that enrolled fewer than 4 participants per month. Noncompliance with study procedures in the first 6 weeks was also associated with subsequent early withdrawal, but dissatisfaction with the spermicide was not. However, many participants without these risk factors withdrew early.

**Conclusions:**

Failure to complete is a major problem in barrier method trials that seriously compromises the interpretation of results. Targeting retention efforts at women at high risk for early withdrawal is not likely to address the problem sufficiently.

## Background

Retention of participants has been a consistent problem in clinical studies of barrier contraceptive methods. For example, in six large studies of condoms, diaphragms, and spermicides conducted in the past decade, more than 30% of the participants failed for reasons other than pregnancy to complete the intended six months or six menstrual cycles of follow-up [1-6] Such high dropout rates seriously compromise the interpretation of trial results.

Issues regarding the design of barrier method studies have become increasingly important to researchers and public health scientists since the onset of the HIV epidemic because of the urgent need for methods to prevent this disease and other sexually transmitted infections. Numerous new barrier contraceptive methods and microbicides are currently in various stages of development and testing. Devising effective approaches to maximize retention in these studies will be critical.

In this analysis, we used data from a large, recently completed randomized trial of the efficacy and safety of five spermicide products to determine whether we could identify specific subgroups of participants who were at particular risk for failure to complete the trial. Our goal was to provide information that might assist in the development of targeted approaches to improve follow-up in future trials.

## Methods

The primary purpose of this randomized trial was to estimate and compare the probability of pregnancy during six months of typical use of five nonoxynol-9 spermicide products. Safety, acceptability, and product use were additional specified outcomes. The trial was conducted at 14 sites in the United States between June 1998 and August 2002. The study was approved by the institutional review boards at each site and at Family Health International. All participants signed written informed consent forms before enrollment.

A full description of the trial procedures has been published previously [7]. In brief, the study enrolled 1536 healthy, sexually active women aged 18–40 years who had no history suggestive of subfecundity, who were at low risk for sexually transmitted infections, and who stated that they were willing to rely on a spermicide as their only contraceptive method for 7 months and to accept a moderate risk of pregnancy. At the enrollment visit, each volunteer had an interview, pelvic examination, Pap smear, wet prep, and urine pregnancy test. After eligibility was established, she completed a self administered questionnaire that included a question about strength of desire to avoid pregnancy. Each eligible participant was randomly assigned to one of the five study spermicide groups. She was given a supply of her assigned spermicide and a diary on which to record relevant information daily throughout the study. Some participants at two centers were enrolled into a substudy to evaluate colposcopic effects of the spermicides. Participants were encouraged but not required to inform their partners about the study except at one center, where the Institutional Review Board required signed consent of the partner.

Follow-up visits were scheduled at 4, 17, and 30 weeks after admission. Each participant was also asked to return to the study site if she wished to discontinue use of the spermicide. At each visit, the participant was interviewed, and a urine pregnancy test was done. At the 4-week and final visits, she completed a seven-page acceptability questionnaire. A pelvic examination was performed at the final visit and at other visits as indicated. Colposcopy substudy participants had a vaginal colposcopy at each follow-up visit. Each participant was asked to do a pregnancy test at home 2, 10, and 23 weeks after admission and to telephone the site with the result. If a participant missed a scheduled contact, study procedures required that staff make at least four attempts to contact her by at least two different modalities (telephone, mail, etc.) If they could not contact her directly, staff were to try to reach her through an alternate contact person identified by the participant at admission. Compensation for completion of all scheduled visits in the primary study ranged from $120 to $400 at the 14 study sites; at most sites, the amount was divided evenly among the separate visits.

In this analysis, we included all randomized participants except for 22 who were discovered to have been pregnant at admission and who therefore contributed no data to the primary analysis. We classified each of the remaining 1514 participants as having completed the study if she considered the spermicide to be her primary contraceptive method for at least 183 days after randomization, or she became pregnant before she stopped relying on it. Otherwise, she was classified as having failed to complete the study. We assigned each participant's last day in the analysis as the earliest of the following dates: the estimated date of fertilization of a pregnancy; the date she was last known to have been relying primarily on the assigned spermicide for contraception; the latest date her pregnancy status could be reliably determined; and 183 days after randomization. These rules were the same as those used in the prior primary pregnancy analyses [7].

We assessed the associations between failure to complete and 17 baseline factors of interest, which were prespecified before the analysis. Among the subset of participants who were in the analysis for at least 6 weeks, we examined the associations between final status category and various factors that characterized their experience during the first 6 weeks in the study. Factors were categorized in part to ensure substantial numbers of participants in each level. Hypotheses about the effects of factors on completion status were tested using chi square tests, Fisher's exact tests, Mantel Haenszel tests. Parameters estimated by multivariable logistic regressions were tested using Wald tests. We included factors in regression models if they were associated with the outcome (alpha<0.10) in bivariable analyses. None of the included factors were highly correlated. In both bivariable and multivariable analyses, a p-value of <0.05 was considered to indicate a significant association.

## Results

Of the 1514 participants in this analysis, 635 (42%) failed to complete the study for reasons other than pregnancy. The proportion who withdrew early at each of the 14 study sites ranged from 17% to 83%. Only 3 centers had completion rates ≥65%. Forty nine participants (8% of those who withdrew) were discontinued by the site investigator because of a concern about their safety (such as increased risk of sexually transmitted infection that would indicate need for condom use, or use of a drug contraindicated in pregnancy that would indicate need for a more effective contraceptive than spermicide alone), staff error, or closure of the trial at the study site (Table 1). Of the 586 who withdrew on their own accord, 382 (65%) did not provide a reason, in most cases because they did not return for a discontinuation visit. The other 204 women reported a variety of reasons; 99 cited complaints that might have been related in some way to the spermicide. Of the other 105 participants, only 31 said that they would like to continue using the spermicide after leaving the study.

**Table 1:** Reasons for early withdrawal

Reason	Participants N = 635	
	
	n	%
Discontinued by site		
Safety concerns	38	6%
Site closure/staff error	11	2%
Participant decision, reason provided*	204	32%
Unwillingness to continue study visits	60	9%
Objections from partner^†^	48	8%
Desire to change contraceptive method^†^	42	7%
Separation from partner	40	6%
Side effects or other medical events^†^	37	6%
Cessation of sexual activity	18	3%
Dissatisfaction with spermicide^†^	8	1%
Distrusted contraceptive efficacy^†^	8	1%
Desire for pregnancy	7	1%
Mistaken suspicion of pregnancy	4	1%
Participant decision, reason unknown	382	60%

*Participants may have provided more than one reason

^†^Considered "related to spermicide" in text

During their time in the analysis, women who failed to complete the study were less compliant with follow-up visits and diary records than women who completed (Table 2). Twenty-one percent of the population (135 participants) contributed no data at all to the analysis after admission.

**Table 2:** Protocol compliance by final status category

	Final status category		p-value^†^
	Completed study	Did not complete study	
	
Number of participants	879 (58%)	635 (42%)	
Median days in analysis per participant	183	32	
Mean % of expected follow-up visits completed*	85%	64%	<.0001
Mean % of expected diary days recorded*	97%	72%	<.0001
Mean % of expected pregnancy tests completed*	95%	97%	0.0001

*The number of expected visits was prorated for each participant considering the total duration of her participation in the analysis

^†^p-value from independent sample t-test.

Of the 17 baseline factors examined separately, nine were associated with significantly increased (p < 0.05) relative risk of failure to complete the trial (Table 3). Factors that did not significantly increase risk included spermicide group, race, educational level, prior spermicide use, strength of desire to avoid pregnancy as reported on the self-administered admission questionnaire, desire for additional children, reason for choosing spermicide as a contraceptive method, and enrollment date relative to notification in 1999 of new data suggesting concern about the possibility that nonoxynol-9 might affect the risk of HIV acquisition. In multivariable analyses including the nine high risk factors and one additional factor (level of schooling, which was marginally associated with withdrawal, p = 0.09), only the associations with young age, unmarried status, frequent intercourse, enrollment at a university center, and enrollment at a center with a lower recruitment rate remained significant.

**Table 3:** Association between baseline factors and failure to complete study

	Total	Did not complete study		Relative Risk (95% confidence interval)
	N	n	%	
	
Age				
≤25 years	660	317	48.0	1.29 (1.15 – 1.45)
> 25 years	854	318	37.2	1
Relationship				
single not living with partner	522	247	47.3	1.21 (1.07 – 1.36)
married or living with partner	992	388	39.1	1
Living children				
None	639	288	45.1	1.14 (1.0 1 – 1.28)
Any	875	347	39.7	1
Baseline coital frequency				
≥8 acts per month	862	389	45.1	1.21 (1.07 – 1.37)
≤7 acts per month	640	239	37.3	1
Geographic region of US				
West	424	215	50.7	1.28 (1.12 – 1.46)
South	460	170	37.0	0.93 (0.80 – 1.09)
Northeast	630	250	39.7	1
Center type				
university*	1069	476	44.5	1.25 (1.08 – 1.44)
other	445	159	35.7	1
Recruitment rate at study site				
≤4 per month	640	301	47.0	1.23 (1.09 – 1.38)
> 4 per month	874	334	38.2	1
Reimbursement rate				
≤$200	462	232	50.2	1.31 (1.16 – 1.47)
>$200	1052	403	38.3	1
Participation in colposcopy study				
No	1381	590	42.7	1.26 (0.99, 1.61)^†^
Yes	133	45	33.8	1

*University centers were defined as those at which participants were seen in a primary university clinic setting. These included: University of Alabama at Birmingham, Birmingham, AL; University of Tennessee at Memphis, Memphis, TN; The University of Texas Health Science Center at San Antonio, San Antonio, TX; Baylor College of Medicine, Houston, TX; Medical University of South Carolina, Charleston, SC; University of Pittsburgh and the Magee-Womens Research Institute, Pittsburgh, PA; University of Pennsylvania Medical Center, Philadelphia, PA; University of Arizona Health Sciences Center, Tucson, AZ; NYU School of Medicine, New York, NY. Other centers included: Johns Hopkins Medical Services Corporation, Baltimore, MD; Vermont Women's Choice Program of Planned Parenthood, Burlington, VT; Eastern Virginia Medical School, Norfolk, VA; Minneapolis Medical Research Foundation, Minneapolis, MN; Planned Parenthood of Central and Northern Arizona, Phoenix, AZ

^†^Although this confidence limit includes 1, the p-value for the association between this factor and early withdrawal was 0.047.

Of the 1095 participants who contributed more than 6 weeks to the analysis, those who in their first 6 weeks were not compliant with follow-up visits, coital diary completion, or use of the spermicide during sex were significantly less likely than others to complete the study (Table 4). However, among the 925 participants who completed a contact during the initial 6 weeks, neither reported complaints nor any measure of satisfaction with the spermicide during the first 6 weeks was associated with increased risk of early withdrawal. We created a single variable to indicate whether or not each subject was "happy" with the spermicide in the first 6 weeks after admission (i.e., she found it acceptable, had no side effect or adverse event, and had a satisfied partner). Women who were "happy" were not significantly less likely than other women to withdraw early.

**Table 4:** Association between early study experience and failure to complete study*

Experience during first 6 weeks	Total	Did not complete study		Relative Risk (95% confidence interval)
	N	n	%	
	
Completed at least one follow-up visit				
yes	925	203	21.9	0.68 (0.53 – 0.87)
no	170	55	32.4	1
Completed at least one pregnancy test within first 4 weeks				
yes	1070	250	23.4	0.73 (0.41 – 1.31)
no	25	8	32.0	1
Provided diary information for each day				
yes	958	215	22.4	0.71 (0.54 – 0.94)
no	137	43	31.4	1
Used spermicide at every coital act				
yes	739	153	20.7	0.70 (0.57 – 0.87)
no	356	105	29.5	1
Of those who completed follow-up visit				
Reported medical complaints				
yes	437	104	23.8	1.17 (0.92 – 1.50)
no	488	99	20.3	1
Disliked spermicide somewhat or a lot				
yes	52	12	23.1	1.05 (0.63 – 1.76)
no	873	191	21.9	1
Distrusted contraceptive efficacy				
yes	209	51	24.4	1.15 (0.87 – 1.52)
no	716	152	21.2	1
Disliked timing of application				
yes	435	96	22.1	1.01 (0.79 – 1.29)
no	490	107	21.8	1
Complained about messiness				
yes	375	76	20.3	0.88 (0.68 – 1.13)
no	550	127	23.1	1
Had problems with insertion				
yes	465	105	22.6	1.06 (0.83 – 1.35)
no	460	98	21.3	1
Reported that partner disliked spermicide				
yes	208	52	25.0	1.18 (0.90 – 1.56)
no	717	151	21.1	1
Happy with spermicide^†^				
yes	408	80	19.6	0.82 (0.64 – 1.05)
no	517	123	23.8	1

*Includes only participants in the analysis for at least 6 weeks

^†^Did not dislike spermicide, had no side effect/AE, and had a satisfied partner

## Discussion and conclusions

In analyzing data from longitudinal studies, researchers commonly assume that the experience of participants who withdraw early, had they stayed in the study, would have been similar to the experience of those who completed. However, this assumption is generally impossible to confirm and is often implausible. If the assumption is false, the study findings may substantially misrepresent the likelihood of the outcome in the study population. If the degree of misrepresentation is not consistent across study groups, comparisons could be seriously biased. Indeed, some expert epidemiologists have suggested that a trial with losses of greater than 20% of the participants "would be unlikely to successfully withstand challenges to its validity" [8,9].

Our study, like other recent barrier contraceptive method studies, did not even approach this standard: 42% of our enrolled participants did not complete the trial. Furthermore, the participants who failed to complete were different in key ways from those who did – they reported significantly more frequent coitus at baseline, and they also were more likely to be younger, unmarried, and poorly compliant with study procedures and method use in the first few weeks after admission. All of these characteristics were associated to some extent with elevated risk of pregnancy in our population [7], which suggests that our high withdrawal rate indeed may have distorted our findings: the pregnancy probabilities that we reported may be underestimates.

Clearly, increased attention to preventing this problem in future studies is imperative. In performing this analysis, our intention was to explore the potential impact of focusing retention efforts on participants with characteristics that are associated with failure to complete. However, although we did find some factors that were significantly associated with early withdrawal, none was highly predictive; that is, many participants without these factors failed to complete the study, and many with these factors did complete. Therefore, applying special efforts only to the high-risk participants would not likely have been sufficient to raise completion rates to desirable levels. In future trials, aggressive follow-up measures should be instituted universally. Such efforts might include assigning individual "case-workers" to participants, using novel means for communicating with the participants, such as pagers, conducting visits at participants' homes or at other locations convenient for them, providing specific reimbursement for expenses such as travel, parking, and child care, or providing extra incentives for completing follow-up. Researchers should be mindful, however, that one downside to some of these approaches is that they might influence participants' use of the study product or other behaviors related to the study outcome, which is detrimental if the goal of the trial is to estimate effectiveness during "typical use" of the product.

In our study, participants who enrolled at study centers where enrollment was slow were at increased risk of failure to complete the study. The reason for this association is unclear. Factors at these centers that hindered enrollment also may have adversely affected participants' interest in remaining in the study. Alternatively, in responding to pressure to hasten recruitment, these centers may have enrolled women who were not good candidates for study completion. This latter possibility emphasizes the need to maintain a careful balance between recruitment and retention goals: rapid recruitment of participants who then drop out of the study is not beneficial to the study as a whole.

The amount of reimbursement promised to our participants was strongly associated with final completion status in the bivariable analysis, but this effect was not significant when adjusted for other factors in our multivariable model. Numerous prior studies have shown that modest monetary incentives (e.g., $20 or less) increase response rates to surveys or short follow-up studies [10,11] Some data also suggest that the value of the incentive matters, although possibly with diminishing returns as the value increases [12,13]. However, the effect of higher levels of compensation in longer trials such as ours has not been rigorously studied. The possibilities that large financial incentives could be coercive, weaken generalizability, or encourage bogus participation are important concerns [14].

We were surprised that several of the factors that we expected would be associated with early withdrawal did not show significant associations in this analysis. When we began this analysis, we presumed that one reason for both slow enrollment and poor follow-up rates in barrier method trials is the relatively poor efficacy of these products: women may consider them to be temporary or backup methods and thus may be unwilling to use them as their sole or primary contraceptive for the 6–12 month duration of these studies. However, in our study, participants who strongly wished to avoid pregnancy or who had completed their desired family size were not more likely than others to drop out, nor were participants who expressed concerns about contraceptive efficacy early in the trial. Furthermore, neither early medical problems nor other complaints about the spermicides were predictive of withdrawal. These findings differ from that of a previous randomized trial of spermicides conducted mostly in developing countries. In that trial, participants who initially liked the assigned product very much were more likely than others subsequently to complete the study and to use the product for a longer period of time after admission [15].

In one respect, the poor retention rate in our study and in other barrier method trials is a result of the design of these studies, which typically call for censoring data (and in most barrier method trials, terminating active follow-up) when participants stop relying on the assigned contraceptive method. This design prohibits a true intent-to-treat analysis and is consequently a potential source of bias. Clearly, retention would be higher if the trials were designed at the outset to follow all subjects for the full intended duration of follow-up, even if they switched contraceptive methods. However, data from participants who are not using the method under study are not necessarily relevant to the efficacy and safety of the method. For the results of these trials to be meaningful, as many subjects as possible must not only complete follow-up but also continue to use the method during the full follow-up period. In our study, almost all the women who gave a reason for withdrawing early either cited problems with the spermicide or indicated that they wished to switch to another method after leaving the study. Our results are consistent with the findings of the 1995 National Survey of Family Growth, which showed that more than 47% of US spermicide users stopped relying on the method within the first 6 months of use [16]. These findings are discouraging: they suggest that even if the retention in the study could be improved by aggressive follow-up techniques, the likelihood of significant extension of method use is low.

Our results suggest that to reduce bias potentially introduced by a large proportion of participants failing to complete the study, future barrier contraceptive method researchers should consider approaches in addition to those directly aimed at tracking and retaining individual participants. For example, both to reduce the burden on participants and to help the study staff maintain focus on follow-up, limiting data collection to critical variables may be appropriate. Complete collection of key data is clearly preferable to inadequate collection of less important data. Reducing the planned duration of follow-up would also certainly reduce withdrawals; although a larger sample size would be needed to provide the desired levels of precision and power, this disadvantage might be overcome if the shorter study were more attractive to potential participants. Given the large proportion of women who stop using the method earlier than 6 months, it is not clear that 6-month pregnancy probabilities are clinically needed anyway. Adding a run-in period to the trial before randomization might be helpful in excluding participants likely to drop out very early after admission, although such an addition might deter enrollment of other women as well, which is also a problem in these trials. Finally, innovative study designs to measure product efficacy should be evaluated. The design proposed by Steiner et al., which compares the one-month pregnancy probability in a relatively small number of women using a contraceptive method to the probability in women using a placebo, offers an alternative to the traditional 6–12 month trial [17]. It showed some promise in a pilot study and is currently being further tested in a study of a new candidate spermicide.

## Competing interests

No authors have any declared interests except the following:

Elizabeth Raymond owns stock in Johnson and Johnson.

Mitchell Creinin serves as a speaker for Ortho.

Alfred Poindexter has had research grants from Columbia Laboratories and serves as speaker for Ortho.

## Authors' contributions

EGR helped design the trial, managed the trial, planned this analysis, and drafted the manuscript.

PLC and BPL helped design the trial and/or this analysis, performed the analysis, and contributed to the manuscript.

JL designed the trial and contributed to the manuscript.

Other authors participated in the design of the trial, conducted the trial, and contributed to the manuscript.

## Pre-publication history

The pre-publication history for this paper can be accessed here:


